# Protein complex formation in methionine chain-elongation and leucine biosynthesis

**DOI:** 10.1038/s41598-021-82790-4

**Published:** 2021-02-10

**Authors:** Li-Qun Chen, Shweta Chhajed, Tong Zhang, Joseph M. Collins, Qiuying Pang, Wenyuan Song, Yan He, Sixue Chen

**Affiliations:** 1 State Key Laboratory of Plant Physiology and Biochemistry, College of Biological Sciences, China Agricultural University, Beijing, China; 2 Department of Biology, Genetics Institute, Plant Molecular & Cellular Biology Program, Interdisciplinary Center for Biotechnology Research, University of Florida, Gainesville, FL USA; 3 Alkali Soil Natural Environmental Science Center, Key Laboratory of Saline−Alkali Vegetation Ecology Restoration in Oil Field, Northeast Forestry University, Harbin, Heilongjiang China; 4 Department of Plant Pathology, University of Florida, Gainesville, FL USA; 5 National Maize Improvement Center of China, Beijing Key Laboratory of Crop Genetic Improvement, China Agricultural University, Beijing, China

**Keywords:** Plant sciences, Secondary metabolism

## Abstract

During the past two decades, glucosinolate (GLS) metabolic pathways have been under extensive studies because of the importance of the specialized metabolites in plant defense against herbivores and pathogens. The studies have led to a nearly complete characterization of biosynthetic genes in the reference plant *Arabidopsis thaliana*. Before methionine incorporation into the core structure of aliphatic GLS, it undergoes chain-elongation through an iterative three-step process recruited from leucine biosynthesis. Although enzymes catalyzing each step of the reaction have been characterized, the regulatory mode is largely unknown. In this study, using three independent approaches, yeast two-hybrid (Y2H), coimmunoprecipitation (Co-IP) and bimolecular fluorescence complementation (BiFC), we uncovered the presence of protein complexes consisting of isopropylmalate isomerase (IPMI) and isopropylmalate dehydrogenase (IPMDH). In addition, simultaneous decreases in both IPMI and IPMDH activities in a *leuc:ipmdh1* double mutants resulted in aggregated changes of GLS profiles compared to either *leuc* or *ipmdh1* single mutants. Although the biological importance of the formation of IPMI and IPMDH protein complexes has not been documented in any organisms, these complexes may represent a new regulatory mechanism of substrate channeling in GLS and/or leucine biosynthesis. Since genes encoding the two enzymes are widely distributed in eukaryotic and prokaryotic genomes, such complexes may have universal significance in the regulation of leucine biosynthesis.

## Introduction

Substrate channeling is the transfer of a metabolic intermediate produced by one enzyme directly to the next enzyme without diffusion into the bulk phase of the reaction environment^1^. This channeling can expedite the metabolic process, prevent the release of unstable intermediates, and keep an intermediate from being taken by competing enzymes in other reactions. To date, only a limited number of cases indicating metabolic channeling via protein − protein interactions have been reported in plants, e.g., the interactions between spermidine synthase 1 (SPDS1) and spermidine synthase 2 (SPDS2), and between SPDS2 and spermine synthase (SPMS) during Arabidopsis polyamines biosynthesis^2^, the Arabidopsis UDP-D-glucose 4-epimerase with a UDP-D-galactose transporter in galactose biosynthesis^3^, the Arabidopsis diaminopelargonic acid aminotransferase (DAPA-AT) and dethiobiotin synthetase (DTBS) in biotin synthesis^4^, the Arabidopsis glutamyl-tRNA reductase (GluTR) and its binding protein (GluBR) complex^5^, and the interaction between strictosidine *β* − D − glucosidase and tetrahydroalstonine synthase in strictosidine agalycone biosynthesis in *Catharanthus roseus*^6^. Recently, tricarboxylic acid cycle enzymes citrate synthase and aconitase were found to interact and channel citrate, and succinate dehydrogenase interacted with fumarase to channel furmarate^7^. Glucosinolates (GLSs) are specialized metabolites derived from amino acids in Brassicales plants. They are known to play important roles in plant defense against insects and pathogens, and their breakdown products have anticarcinogenic activities^8,9^. Based on the amino acid precursor, GLSs can be grouped into aromatic, indolic and aliphatic GLSs. Aliphatic GLSs are derived from chain − elongated methionine (Met) substrates after a repeated cycle with three enzymatic steps, known to be recruited from leucine (Leu) biosynthetic pathway during evolution^10^. These two pathways of glucosinolate and Leu biosynthesis have homologous enzymes at each step, which are either shared between the two pathways or specialized in one of the pathways (Supplemental Fig. 1). At the condensation step, the two methylthioalkylmalate synthases (MAM1 and MAM3) in GLS biosynthesis show 60% identity in amino acid sequence with the two isopropylmalate synthases (IPMS1 and IPMS2) in Leu biosynthesis^11,12^. The isomerization reaction is catalyzed by a heterodimeric enzyme isopropylmalate isomerase (IPMI) consisting of a large subunit encoded by a single gene (*LeuC*) and a small subunit encoded by one of the three genes (*LeuD1*, *LeuD2* and *LeuD3*). Compared to the dual function of LeuC in both pathways, the functions of the LeuDs are specialized. LeuD1 and LeuD2 are redundantly involved in Met chain − elongation of GLS biosynthesis, whereas LeuD3 functions in Leu biosynthesis^13–16^. Lastly, at the decarboxylation step, isopropylmalate dehydrogenase 1 (IPMDH1) has a predominant role in Met chain − elongation, while IPMDH2 and IPMDH3 play redundant roles in Leu biosynthesis^14,17,18^.

Compared to the above well-characterized enzymes, little is known about the mechanisms of metabolic regulation in the two pathways. The only recognized mechanism is the allosteric control of IPMS activity by the final product, Leu, to maintain Leu homeostasis in planta. We hypothesize that protein interactions play a regulatory role in channeling Met elongation and/or Leu biosynthesis. In this work, we have conducted screening of protein–protein interactions between all the identified proteins involved in Met chain − elongation pathway using yeast two − hybrid (Y2H), identified novel interactions between IPMIs and IPMDHs, and confirmed in vivo interaction using co-immunoprecipitation (Co-IP) and bimolecular fluorescence complementation (BIFC). The biological importance of the newly identified protein–protein interactions is discussed. The complex formation may represent a new regulatory mechanism controlling Met chain-elongation and/or Leu biosynthesis.

## Results

### Y2H interaction screening of proteins involved in Met chain-elongation

**Figure 1:**
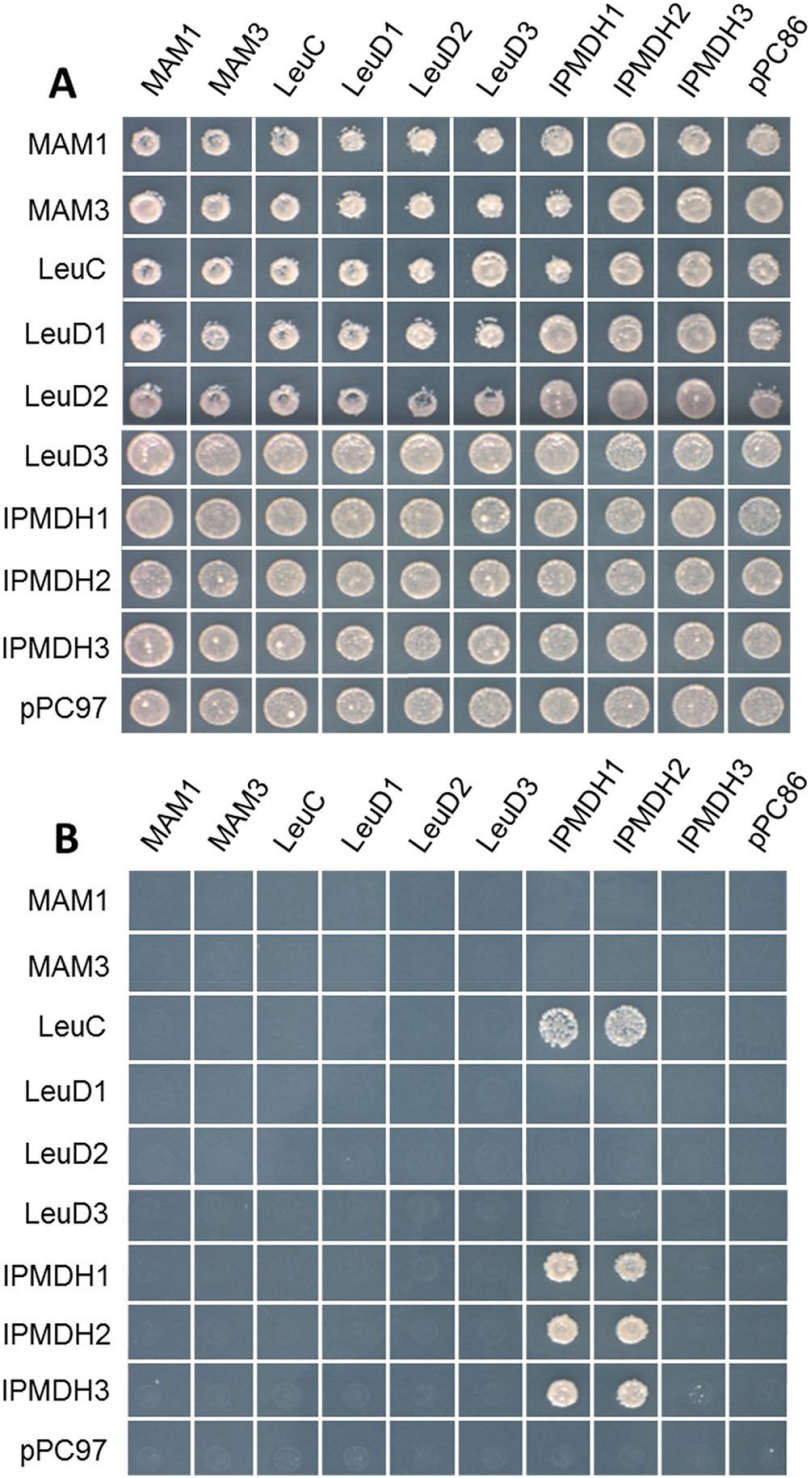
Pair-wise Y2H screening for interactions among nine genes involved in Met chain-elongation pathway. (**A**) Combinations of proteins used in Y2H assay. Transformed yeast with indicated plasmid combinations was plated onto SD media lacking Trp and Leu. Plasmids pPC97 and pPC86 carry GAL4DNA binding domain and GAL4 activation domain, respectively. (**B**) Transformed yeast with indicated plasmid combinations was plated onto SD media media lacking Trp, Leu, His and containing 1 mM 3-Amino-1,2,4-Trizaole (3AT).

According to the previous studies, there are six genes involved in the Met chain − elongation pathway of aliphatic GLS, including *MAM1*, *MAM3*, *LeuC*, *LeuD1*, *LeuD2*, and *IPMDH1*^12–16,18–21^ (Supplemental Figure S1). To clarify the inconsistent naming of these genes from different research groups (e.g., He et al., 2010 versus Imhof 2014), all the genes used in the study (including *LeuD3*, *IPMDH2* and *IPMDH3*) were listed and annotated in Supplemental Table 1. To screen for potential protein–protein interactions among the genes, a pair-wise Y2H assay was conducted. Different protein–protein combinations are illustrated in Fig. 1A. When culturing the yeast cells transformed with the gene-containing plasmids in the yeast synthetic defined (SD) plate with tryptophan (Trp) and Leu (Leucine) dropouts, all the combinations can grow into colonies, indicative of successful yeast transformation (Fig. 1A). Cells capable of growing on the triple − deficient medium with 3AT indicate positive protein interactions. Clearly, IPMDH1 and IPMDH2 formed homo − and hetero − dimers, and they also formed heterodimers with IPMDH3 (Fig. 1B). The homo − dimer formation in IPMDH2 has been observed in our study of protein crystal structure^20^. In contrast, faint growth of yeast colonies suggested that the IPMDH3 homodimer was barely formed (Fig. 1B). IPMI is a heterodimeric enzyme and the interaction between the large and small subunits in Arabidopsis has been confirmed by coimmunoprecipitation (Co-IP) experiment in our previous study^15^. However, no interactions could be found between LeuC and any of the LeuDs in our Y2H experiments (Fig. 1B). This could be explained by the fact that the formation of IPMI complex may require other binding cofactors, such as an Fe-S cluster, which was discussed in our previous study^15^. Strikingly, the interaction between LeuC and IPMDH1 or IPMDH2 (Fig. 1B) highlights the potential existence of a new protein complex consisted of IPMI and IPMDHs. MAM1 or MAM3 did not exhibit any detectable interactions with the other proteins, suggesting that the MAM synthases may not be part of the protein complex.

### Interaction of IPMI and IPMDHs in planta confirmed by Co-IP

**Figure 2:**
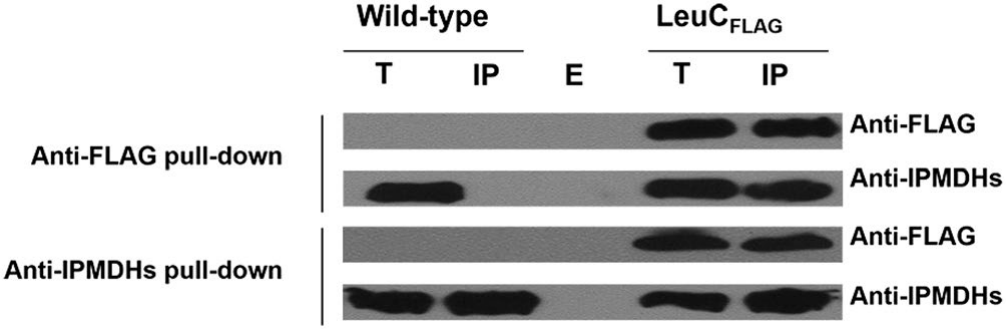
Physical interaction between LeuC and IPMDH in vivo. Chloroplastic proteins prepared from wild-type and *LeuC*-*FLAG* transgenic plants were incubated with anti-FLAG antibody-coupled agarose or anti-IPMDH antibody-coupled agarose. The immunoprecipitate (IP) and the total protein (T)were separated on SDS-gels and subjected to Western blot analysis with anti-FLAG and anti-IPMDH antibody, respectively. E, IP control using empty beads.

To confirm the interaction between LeuC and IPMDHs identified in the Y2H system, in vivo Co-IP approach was done using a transgenic plant harboring a FLAG-tagged LeuC generated in a previous study^15^. As shown in Fig. 2, IPMDH could be readily detected when co-immunoprecipitated alongside LeuC-FLAG, but not from wild-type plants, indicating that IPMDHs interact with LeuC in planta. This result was further corroborated by a reciprocal immunoprecipitation experiment where the LeuC-FLAG signal could be detected after Co-IP using the anti-IPMDH antibody (Fig. 2). These results clearly demonstrate that there is physical interaction between IPMI and IPMDHs in planta. However, because the anti-IPMDH antibody recognizes all three IPMDHs, it is not certain which IPMDHs actually interact with LeuC in planta (Supplemental Fig. 2).

### Bimolecular fluorescence complementation (BiFC)

**Figure 3:**
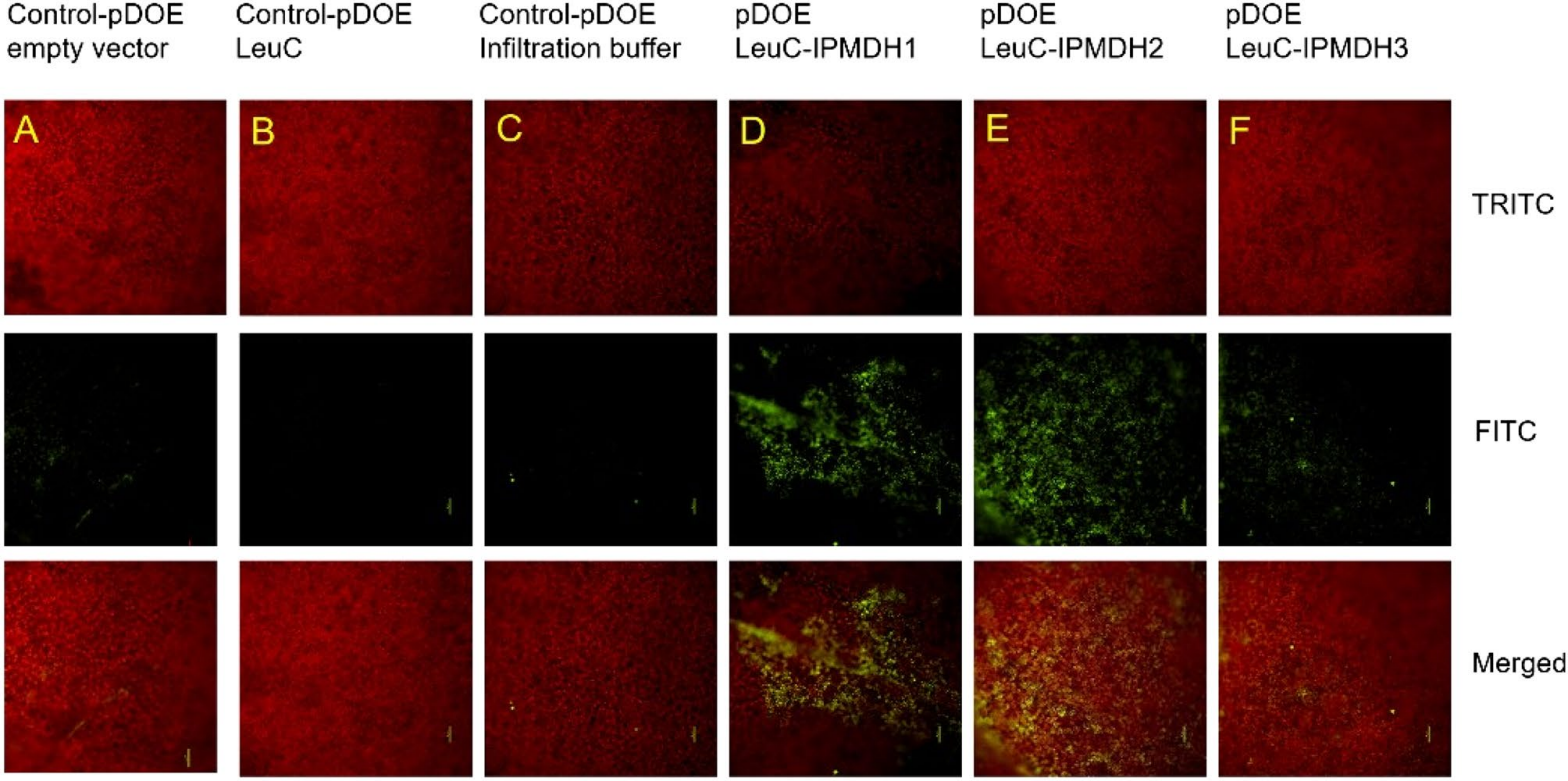
BiFC of the interaction between LeuC and IPMDHs using the pDOE–mVenus210 system. *Nicotiana benthamiana* leaves were agroinfiltrated at an optical density of 0.4 and imaged at Day 3. Scale bars = 100 µm. The chlorophyll autofluorescence was detected with the TRITC filter cube (excitation filter: 527.5–565, barrier filter: 577.7–632.5) (top panel), the mVENUS fluorescence was detected using the FITC filter cube (excitation filter: 465–495, barrier filter: 515–155) (middle panel), and the merged images of TRITC and FITC at the bottom panel. (**A**) *NmVen210–X:CVen210* empty vector showing background signal; (**B**) *LeuC:NmVen210–X:CVen210* vector showing background signal; (**C**) Infiltration buffer control showing background signal; (**D**) LeuC:NmVen210 interacting with IPMDH1:CVen210; (**E**) LeuC:NmVen210 interacting with IPMDH2:CVen210; (F) Weak interaction between LeuC:NmVen210 and IPMDH3:CVen210.

To determine which IPMDHs interact with LeuC in planta, we used BiFC to test the direct interactions of the proteins. As shown in Fig. 3, LeuC clearly showed strong interactions with IPMDH1 and IPMDH2. Its interaction with IPMDH3 was much weaker than with IPMDH1 and IPMDH2. These results corroborate the Y2H and Co-IP results. They together provide strong evidence for complex formation between LeuC and IPMDHs, especially IPMDH1 and IPMDH2. The puncta patterns of the BiFC signals might be attributed to the 35S promoter used.

### GLS profile in *leuc* and *ipmdh1* double mutants

**Figure 4:**
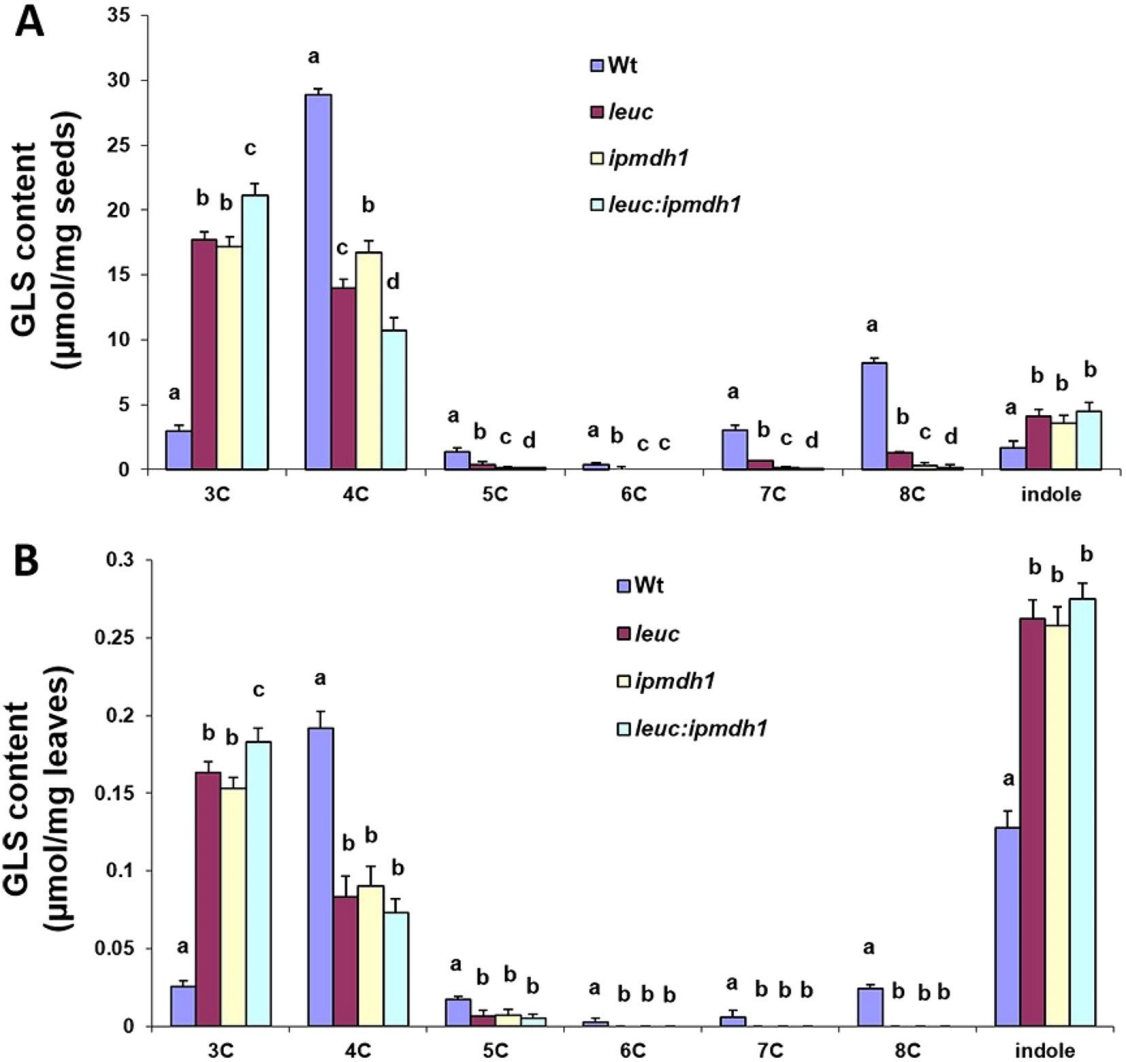
GLS pattern in wild-type (Wt), *leuc*, *ipmdh1* and *leuc*:*ipmdh1* mutants. Aliphatic GLSs are grouped according to their chain length (C3–C8), and indole GLS were summed as one group. GLS concentrations are given in µmol/mg ± standard deviation of three independent replicates. Statistical significant differences (F-test, *P* < 0.05) are indicated by different letters. (**A**) Seeds. (**B**) Leaves.

It has been demonstrated that LeuC plays dual roles in both GLS and Leu biosynthesis, however, the three IPMDH members are specialized, with IPMDH1 involved in GLS metabolism and IPMDH2 and IPMDH3 redundantly participating in Leu biosynthesis. The disruption of LeuC and IPMDH1 led to almost identical changes in the GLS profiles^15,17^, demonstrating that potential metabolic flux via Met chain-elongation pathway was affected in a similar manner under the conditions of decreased IPMI and IPMDH activities. To test the effect of simultaneous disruption of both LeuC and IPMDH1 on GLS biosynthesis, the double-mutant of *leuc*:*ipmdh1* was generated and GLS profiles were analyzed in both seeds and leaves. As shown in Fig. 4, in the double mutant GLS profile was different from the *leuc* and *ipmdh1* single mutants with further enhanced 3C abundance and decrease in long-chain aliphatic GLS in both seeds and leaves. This result suggests that the causal blockage of metabolic flux into long-chain reaction was exaggerated in the double mutant, and the concurrent reduction in the IPMI and IPMDH activities has marked impact on the Met chain-elongation pathway.

## Discussion

Substrate channeling, also called metabolic channeling, is a mechanism to accelerate the passing of intermediary metabolic products by connecting two enzymes together through direct protein interactions^1^ When several sequential enzymes of a metabolic pathway are channeled together, the complex is called a metabolon^2,7^. There are only a few cases of substrate channeling known to occur in plants. SPDS1 and SPMS, two enzymes involved in the last steps of polyamine biosynthesis in Arabidopsis, were demonstrated to form heterodimer through in vitro pull-down and in vivo Co-IP analysis^2^. DAPA-AT and DTBS catalyze the antepenultimate and the penultimate steps, respectively, of plant biotin synthesis. The biochemical and kinetic characterizations have demonstrated that the DAPA intermediate is able to be transferred from the DAPA-AT active site to the DTBS active site^4^. In plant tetrapyrrole biosynthesis, the dimeric GluTR binding protein (GluBP) can symmetrically bind to the catalytic domains of glutamyl-tRNA reductase (GluTR) dimer to stimulate GluTR catalytic efficiency by triggering a substantial conformational change^5^.

Met chain-elongation pathway consists of a repeated cycle with three enzymatic steps, which is known to be recruited from Leu biosynthesis during evolution^10^. Several studies in the past few years have led to the identification of all the genes involved in Met chain-elongation and Leu biosynthesis^11–16,18–20^. However, little is known about the regulatory mechanisms controlling enzymatic activities and how the enzymes affect metabolic flux in both pathways. The allosteric feedback regulation by Leu is the only known mechanism to regulate Leu biosynthesis, and it is achieved by allosteric inhibition of IPMS activity after binding with the final product Leu^22^. Although our previous study has identified that IPMDH1 is a redox sensitive protein, it is still unclear about the role of this modification in controlling Met chain-elongation pathway in planta^17^. Whether additional regulatory mechanisms occur to regulate Leu biosynthesis is currently unknown.

The stereospecific isomerization of 2-isopropyl-L-malate to 3-isopropyl-L-malate is catalyzed by IPMI, which exists as a monomeric protein with two distinct domains in fungi, but the two domains are expressed as separate protein subunits that produce a heterodimeric enzyme in bacteria and plants^23^. In our Y2H assay, we failed to observe the interaction between IPMI large subunit and small subunit, which might be explained by the requirement of other cofactors for complex formation, such as Fe_4_S_4_^24^.The dimeric formation between IPMDHs was further confirmed in the Y2H assay, which is consistent with our previous protein crystal structure study^20^.

Interestingly, we identified an unprecedented interaction of the IPMI large subunit (LeuC) with IPMDH1 or IPMDH2, and a weaker interaction with IPMDH3 in both Y2H and BiFC experiments. From the Co-IP data, it can be concluded that LeuC interacts with IPMDHs. However, because the antibody used recognized all three IPMDHs, BiFC was used to further validate the interaction of LeuC with both IPMDH1 and IPMDH2. Thus, the result indirectly supports our hypothesis that regulation of GLS biosynthesis may occur through substrate channeling. IPMDH1 is the major enzyme that participates in Met chain-elongation pathway, whereas IPMDH2 and IPMDH3 are functionally redundant in Leu biosynthesis with IPMDH3 playing a larger role than IPMDH2. Thus, in this scenario, the protein complex of IPMI and IPMDH may be mainly associated with the Met chain-elongation pathway. Based on this assumption, we speculate that substrate channeling may facilitate the flux in Met chain-elongation reactions either to accelerate the metabolic process or to prevent the release of toxic intermediates generated by the Met chain-elongation reactions in the cell. Alternatively, specific formation of the complex between IPMI and IPMDH1 may promote sufficient IPMI product being channeled into Met chain-elongation pathway. This result is corroborated by the undetectable IPMI product in wild type plants, which indicates efficient turnover (Supplemental Fig. 3)^25^. No matter which of the above scenario exists, previous results showing that single IPMI and IPMDH1 mutations have similar GLS profiles support the interaction between IPMI and IPMDH1 in the Met chain-elongation pathway^15,17^. Interestingly, in the double *leuc*:*ipmdh1* mutant, the alteration of the GLS pattern was further exaggerated, which suggests redundant pathways exist that minorly contribute to the substrate flux. In fact, it is known IPMDH2 and IPMDH3 have residual activity toward glucosinolate substrates^17,20,25^. Furthermore, the result can be explained by our finding that IPMI and IPMDH exist in a protein complex. The decrease in either IPMI or IPMDH results in partial perturbation to the function of the complex, whereas simultaneous reduction of the two enzymes has a larger effect on stoichiometric occurrence of the protein complex. In summary, discovery of the IPMI and IPMDH1 complex formation suggests that it is possible that metabolic channeling may play a role in Met chain-elongation, and it warrants further research to identify the glucosinolate metabolon. As the Leu biosynthetic pathway is widely present and conserved in both eukaryotic and prokaryotic organisms, the finding of a protein complex consisting of IPMI and IPMDH also has broader implications regarding regulation of Leu biosynthesis.

## Methods

### Plant materials

*Arabidopsis thaliana* accession Columbia (Col0) seeds and Salk mutant lines *Salk* 29510 (leuc) and *Salk* 634230 (ipmdh1) were obtained from the Arabidopsis Biological Resource Center (Ohio, USA). The *leuc*:*ipmdh1* double mutant was generated through crossing the two single mutants. Seed sterilization, germination, plant growth and genotyping were conducted as previously described^15,17^. *Nicotiana benthamiana* plants were grown in the same growth chamber as the Arabidopsis under a photosynthetic flux of 140 µmol photons m^−2^ s^−1^ with a photoperiod of 8 h light at 22 °C and 16 h dark at 18 °C. Eight-week-old plants were used for Bimolecular fluorescence complementation (BiFC) experiments.

### Generation of LeuCFLAG transgenic Arabidopsis plants

The cDNA fragment encoding LeuC^15^ was cloned into a pCAMBIA1300FLAG vector^26^. The resultant construct was transformed into *Agrobacterium tumefaciens* strain C58C1 by electroporation. The Agrobacterium harboring the construct was cultured overnight in LB medium with 50 mg/L kanamycin and 25 mg/L rifampicin to reach an OD600 of 0.6. The bacteria were then used to transform the LeuC-FLAG construct into the wild-type *A. thaliana* Col0 plants using a floral dip method of Agrobacterium-mediated transformation^27^.

### Yeast two-hybrid (Y2H) assay

Nine full-length coding sequences of the genes involved in Met chain-elongation pathway, including *MAM1*^19^, *MAM3*^12^, LeuC^15^, *Leu D1*^15^, *Leu D2*^15^ and *IPMDH1*^17^ and Leu biosynthesis genes *Leu D3*^15^, *IPMDH2*^18,20^ and *IPMDH3*^18,20^ were PCR amplified from 4-week old Arabidopsis leaf mRNA using the SUPERSCRIPT III First-Strand Synthesis System (Invitrogen). The PCR conditions were described in the aforementioned references. The IN-FUSION Cloning (Clontech) kit was utilized to clone all the genes following the manufacturer’s instructions. Y2H assays were based on constructs utilizing vectors pPC97 carrying GAL4DNA binding domain and pPC86 carrying GAL4 activation domain. Inserts were cloned between the NdeI/PstI and NdeI/XhoI restriction sites, respectively.

Each pair of constructs listed in Fig. 1A was co-transformed into the yeast strain CG1945, as previously described^28^. Cells growing on a synthetic defined (SD) double-deficient medium (SD/-Trp-Leu) were transferred onto a triple-deficient medium (SD/-Trp-Leu-His) that also contained 1 mM 3-Amino-1,2,4-Triazole (3AT). Cells capable of growing on this triple-deficient medium with 3AT indicate positive protein interactions.

### Chloroplast fractionation, Co-IP and Western blotting

Chloroplast isolation and fractionation from the wild type Arabidopsis and LeuC-FLAG transgenic plants were conducted as previously described^17^. Total protein was extracted from the purified stroma fraction using an immunoprecipitation lysis/binding buffer (50 mM Tris–HCl, pH 7.5, 150 mM NaCl, 10 mM MgCl_2_, 1 mM EDTA, 1 mM EGTA, 0.5% Triton, 0.5% NP-40, 1 mM dithiothreitol (DTT), 10% glycerol, 1 mM phenylmethyl sulfonyl-fluoride (PMSF) and 1 × protease inhibitor cocktail). The extract was centrifuged at 14,000 rpm for 10 min at 4 °C, and the protein concentration in the supernatant was determined using Bradford assay (Bio-Rad Inc., CA, USA). A total of 2 mg of protein was incubated with 3 mg of polyclonal anti-AtIPMDH antibody (produced by Cocalico Biologicals Inc., PA, USA) for 3 h at 4 °C, followed by precipitation with protein A/G Sepharose beads for 2 h. Alternatively, aliquots of 50 µl of anti-FLAG antibody-conjugated agarose beads (Sigma-Aldrich, MO, USA) were mixed with 1 mg of the total protein extracts for 3 h at 4 °C. After washing the beads four times with lysis/binding buffer, the affinity-bound proteins were eluted by boiling for 5 min in 2 × Laemmli SDS sample buffer before loading onto a 12% SDS–polyacrylamide gel. Western blot was conducted as described^17^. Proteins immunoprecipitated with the anti-IPMDH antibody were probed with the anti-FLAG antibody (1:10,000) and vice versa with the anti-IPMDH antibody (1:2000).

### Bimolecular fluorescence complementation (BiFC) analysis

In-fusion cloning was used to build the constructs for BiFC experiments. The *LeuC* coding sequence was first introduced into the multiple cloning site (MCS) upstream of NmVen210 via homologous recombination with NcoI-linearized pDOE-01. The resultant *LeuC-NmVen210* vector was then linearized with KflI to incorporate *IPMDH1*, *IPMDH2*, or *IPMDH3* coding sequences at the upstream of CVen210. Three negative controls were used in this experiment: (a) Vector control (*NmVen210–X: CVen210*); (b) *LeuC:NmVen210–X:CVen210* parent vector; (c) Infiltration control (50 mM MES, pH 5.6, 10 mM MgCl_2_ and 150 µM acetosyringone). Experimental samples used were: (d) *LeuC:NmVen210* with *IPMDH1:CVen210*; (e) *LeuC:NmVen210* with *IPMDH2:CVen210*; (f) *LeuC:NmVen210* with *IPMDH3:CVen210*.

Agrobacterium with different constructs were cultured overnight in LB (Luria − Bertani) medium with 50 mg/L kanamycin and 25 mg/L rifampicin and 150 µM acetosyringone to an OD600 of 0.8 with continuous shaking at 28 °C. The bacteria were then pelleted and suspended in the infiltration solution at OD600 of 0.4^29,30^. The bacteria were infiltrated into the *N. benthamiana* plant leaves with a needleless syringe. The leaves were analyzed for the BiFC signal 3 days post infection (DPI) using a Nikon Eclipse Ni-E (NIKON) microscope equipped with an Andor Zyla 4.2p sCMOS camera (Oxford Instruments), an X-CITE 120 LED light source (Excelitas Technologies) and controlled with the NIS-elements software (NIKON). mVENUS fluorescence was detected using the FITC filter cube (excitation filter: 465–495, barrier filter: 515–155) and chlorophyll autofluorescence was detected with the TRITC filter cube (excitation filter: 527.5–565, barrier filter: 577.7–632.5).

### GLS profile analysis

Arabidopsis wild type and mutants (*leuc*, *ipmdh1* and *leuc*:*ipmdh1*) were grown side-by-side in a growth chamber, and 4-week-old rosette leaves (100 mg) and seeds (20 mg) were used for GLS analysis. GLSs were analyzed using a Shimadzu HPLC-4000QTRAP MS/MS system^17^ with benzylglucosinolate as internal standard. Both wild type and mutants were run on the same day with blanks between each sample.

## Supplementary Information

Supplementary Information.
