# Supplementary material for: Protein complex formation in methionine chain-elongation and leucine biosynthesis
Source: Sci Rep. 2021 Feb 10;11:3524. doi: 10.1038/s41598-021-82790-4 (PMC7876033; doi:10.1038/s41598-021-82790-4)

**Protein complex formation in methionine chain-elongation and leucine biosynthesis**

# Li-Qun Chen^1,2^, Shweta Chhajed^2^, Tong Zhang^2^, Joseph M Collins^2^, Qiuying Pang^2,3^, Wenyuan Song^4^, Yan He^2,5, *^, and Sixue Chen^2,*^

^1^State Key Laboratory of Plant Physiology and Biochemistry, College of Biological Sciences, China Agricultural University, Beijing, China

^2^Department of Biology, Genetics Institute, Plant Molecular & Cellular Biology Program, Interdisciplinary Center for Biotechnology Research, University of Florida, Gainesville, Florida, USA

^3^Alkali Soil Natural Environmental Science Center, Key Laboratory of Saline−alkali Vegetation Ecology Restoration in Oil Field, Northeast Forestry University, Harbin, Heilongjiang, China

^4^Department of Plant Pathology, University of Florida, Gainesville, Florida, USA

^5^National Maize Improvement Center of China, Beijing Key Laboratory of Crop Genetic Improvement, China Agricultural University, Beijing, China

^*^Correspondence: Sixue Chen, E−mail: [schen@ufl.edu](mailto:schen@ufl.edu); Yan He, Email: [yh352@cau.edu.cn](mailto:yh352@cau.edu.cn)


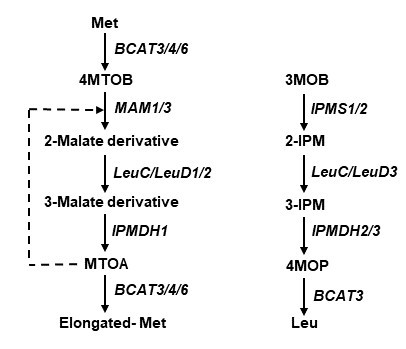


**Supplemental Figure S1.** Met chain-elongation process in aliphatic GLS (left panel) and the late steps of Leu biosynthesis (right panel). The dotted line indicates the repeated cycles adding up to six methylene group. Abbreviations of enzymes are given in the text and abbreviations of metabolites are: Met, methionine; 4MTOB, 4-methylthio-2-oxobutanoate; MTOA, 5-Methythio-2-oxopentanoate; 3MOB, 3-methyl-2-oxobutanoate; 2-IPM, 2-isopropylmalate; 3-IPM, 3-isopropylmalate; 4MOP, 4-methyl-2-oxopentanoate.


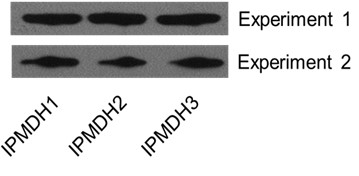


**Supplemental Figure S2.** Specificity of the anti-IPMDH antibody towards three different IPMDH proteins determined by two independent Western blot experiments. A total of 0.5 *µ*g purified recombinant protein of each IPMDH was used in this experiment.


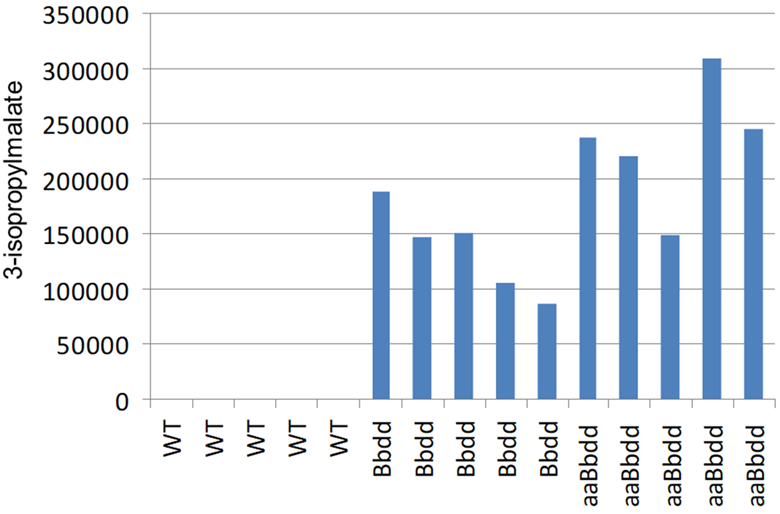


**Supplemental Figure S3.** Levels of 3-isopropylmalate in wild type (WT), *Bbdd* mutant (B, *IPMDH2*; b, *ipmdh2*; d, *ipmdh3*) and *aaBbdd* mutant (a, *ipmdh1*; B, *IPMDH2*; b, *ipmdh2*; d, *ipmdh3*). Please refer to reference 25 for methods and detailed information about the mutants.

**Supplemental Table S1.** Gene annotation with unified names used in this study

| Gene ID | Annotation | Nomenclature in this study | Gene names used in other studies |
| --- | --- | --- | --- |
| AT5G23010 | methylthioalkylmalate synthase 1 | *MAM1* | *MAM1*^19^ |
| AT5G23020 | methylthioalkylmalate synthase 2 | *MAM2* | *MAM2*^12^ |
| AT4G13430 | isopropylmalate isomerase large subunit | *LeuC* | *IPMI LSU1*^13^ |
| AT2G43100 | isopropylmalate isomerase small subunit 1 | *LeuD1* | *IPMI SSU2*13*,*16 |
| AT3G58990 | isopropylmalate isomerase small subunit 2 | *LeuD2* | *IPMI SSU3*13*,*16 |
| AT2G43090 | isopropylmalate isomerase small subunit 3 | *LeuD3* | *IPMI SSU1*13*,*16 |
| AT1G31180 | isopropylmalate dehydrogenase 1 | *IPMDH1* | *IMD1*^14^ |
| AT1G80560 | isopropylmalate dehydrogenase 2 | *IPMDH2* | *IMD2*^14^ |
| AT5G14200 | isopropylmalate dehydrogenase 3 | *IPMDH3* | *IMD3*^14^ |

**Supplemental documentation of original images for Western blot data shown in Figure 2 and Supplemental Figure S2.**

**
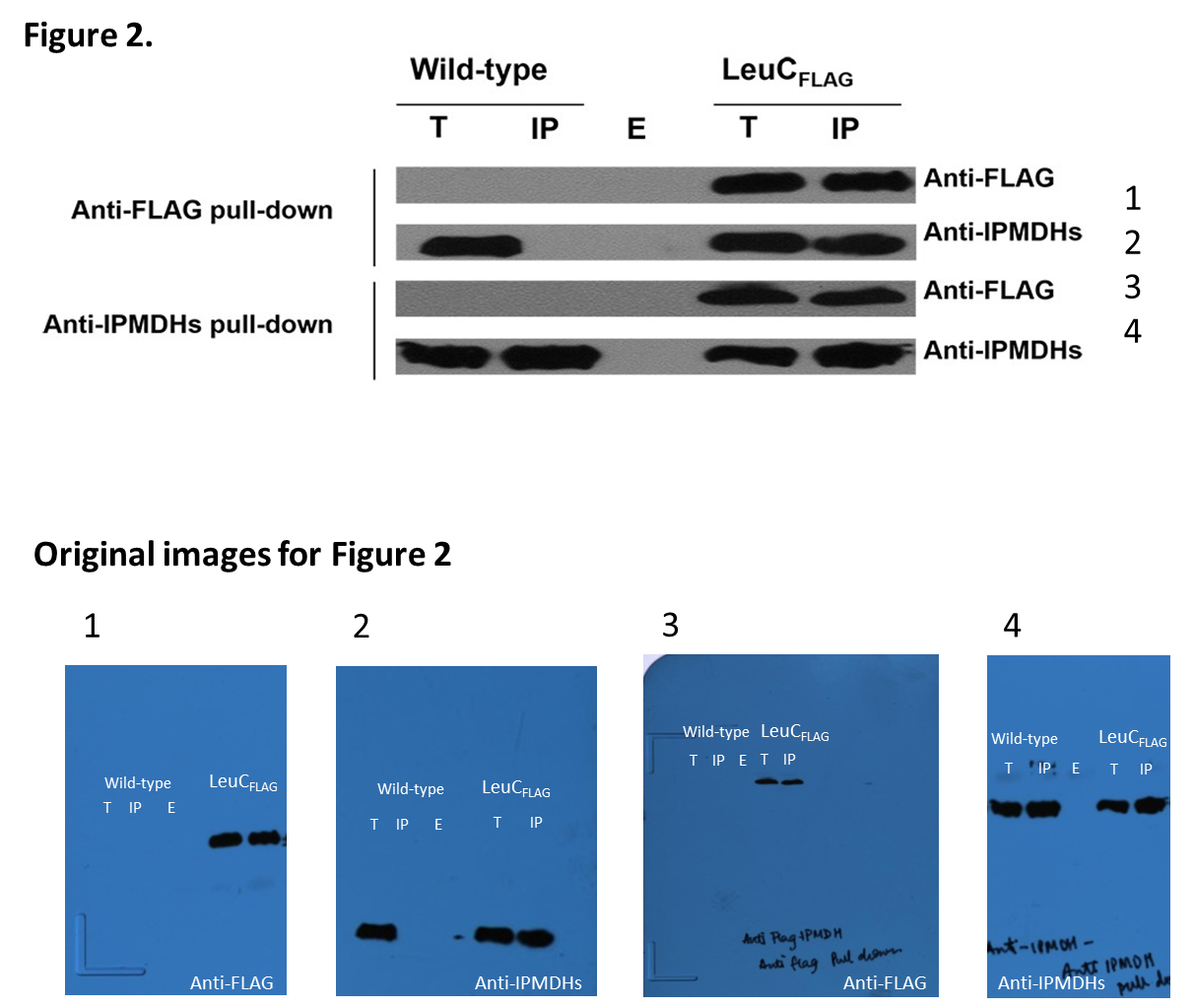
**


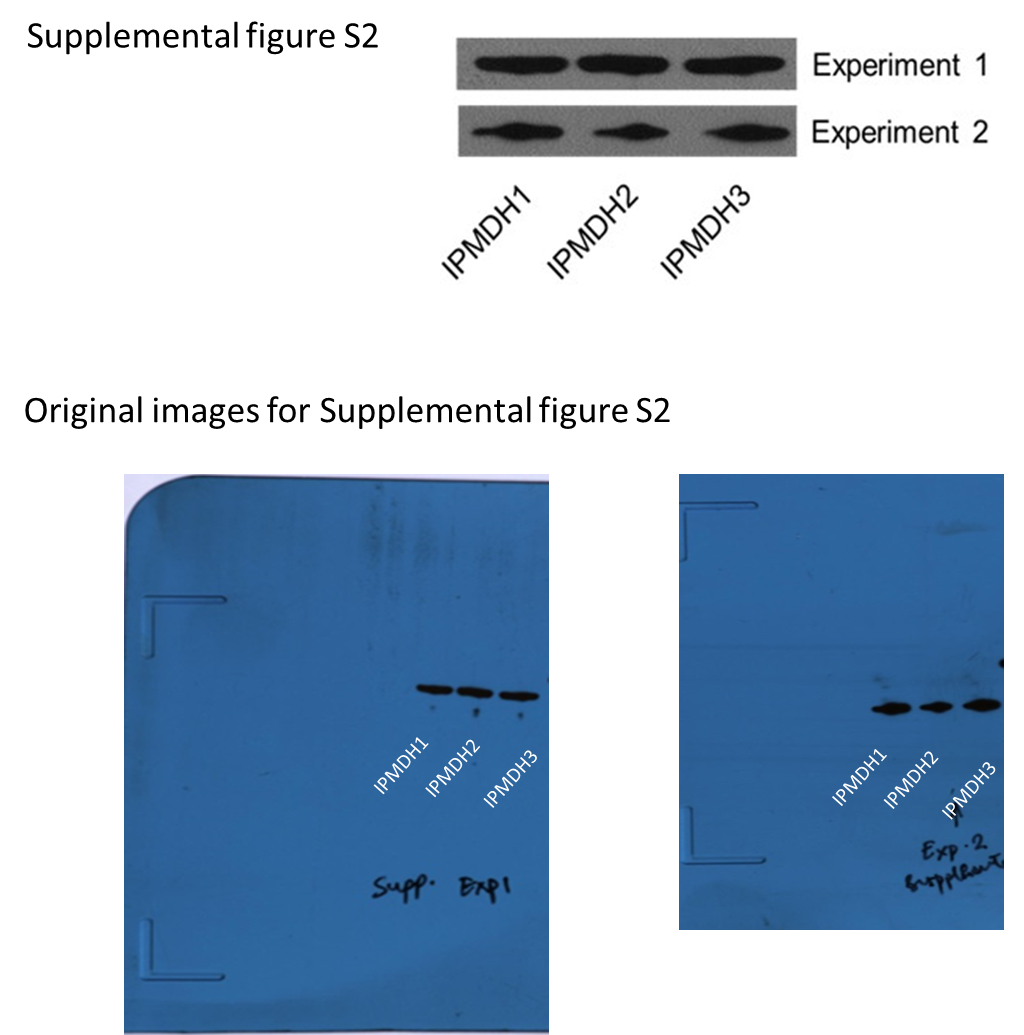

Supplement: Supplementary file 1 — Supplementary Information. [file 41598_2021_82790_MOESM1_ESM.docx]
